# CervSpineNet: a hybrid deep learning-based approach for the segmentation of cervical spinous processes

**DOI:** 10.3389/fbioe.2025.1733689

**Published:** 2026-01-19

**Authors:** Jay Sunil Sawant, Lama Moukheiber, Anupama Nair, Anubha Mahajan, Jaehui Byun, Ishwarya Pichaimani, Sangwook T. Yoon, Christopher T. Martin, Cassie S. Mitchell

**Affiliations:** 1 Laboratory for Pathology Dynamics, Department of Biomedical Engineering, Georgia Institute of Technology and Emory University, Atlanta, GA, United States; 2 Center for Machine Learning, Georgia Institute of Technology, Atlanta, GA, United States; 3 School of Computer Science, Georgia Institute of Technology, Atlanta, GA, United States; 4 Department of Orthopedic Surgery, Emory University, Atlanta, GA, United States; 5 Department of Orthopedic Surgery, University of Minnesota, Minneapolis, MN, United States

**Keywords:** artificial intelligence, automated musculoskeletal landmark detection, cervical spine segmentation, cervical spinous process dataset, deep learning in radiology, hybrid transformer–CNN architecture, machine learning, radiology workflow automation

## Abstract

**Introduction:**

Accurate segmentation of cervical spinous processes on lateral X-rays is essential for reliable anatomical landmarking, surgical planning, and longitudinal assessment of spinal deformity. However, no publicly available dataset provides pixel-level annotations of these structures, and manual delineation remains time-consuming and operator dependent. To address this gap, we curated an expert-labeled dataset of 500 cervical spine radiographs and developed CervSpineNet, a hybrid deep learning framework for automated spinous process segmentation.

**Methods:**

CervSpineNet integrates a transformer-based encoder to capture global anatomical context with a lightweight convolutional decoder to refine local boundaries. Training used a compound loss function that combines Dice, Focal Tversky, Hausdorff distance transform, and Structural Similarity (SSIM) terms to jointly optimize region overlap, class balance, structural fidelity, and boundary accuracy. The model was trained and evaluated on three dataset variants: original images, contrast-enhanced images using CLAHE, and augmented images. Performance was benchmarked against four baselines: U-Net, DeepLabV3+, the Segment Anything Model (SAM), and a text-guided SegFormer.

**Results:**

Across all experimental settings, CervSpineNet consistently outperformed competing methods, achieving mean Dice coefficients above 0.93, IoU values above 0.87, and SSIM above 0.98, with substantially lower HD95 distances. The model demonstrated strong agreement with ground truth, with global MAE ≈ 0.005, and maintained efficient inference times of 5–10 seconds per image. With a compact footprint of approximately 345 MB, CervSpineNet runs on standard clinical hardware and reduces manual annotation time by about 96%.

**Discussion:**

These results indicate that combining transformer-driven global context with convolutional boundary refinement enables robust and reproducible spinous process segmentation on lateral cervical radiographs. By pairing an expert-annotated dataset with a high-performing, computationally efficient model, this work provides a scalable foundation for AI-assisted cervical spine analysis, supporting rapid segmentation for surgical evaluation, deformity monitoring, and large-scale retrospective studies in both research and clinical practice.

## 1 Introduction

The cervical spine—the upper segment of the vertebral column comprising seven articulating vertebrae (C1–C7)—plays a critical biomechanical and protective role. The craniocervical junction, formed by the atlas (C1) and axis (C2), enables head rotation and flexion–extension, while the subaxial spine (C3–C7) provides load-bearing stability and supports the weight of the head (Kaiser et al., 2025). Each vertebra features a posterior bony prominence called the spinous process, whose morphology varies substantially across levels and between individuals (Cramer, 2014). These spinous processes serve as essential anatomic landmarks for vertebral identification, muscle attachment, and radiologic orientation. Their visibility and geometry make them key indicators in diagnostic imaging, surgical navigation, and postoperative evaluation.

Accurate assessment of the cervical spinous processes is clinically significant in several contexts. Avulsion injuries such as clay-shoveler’s fractures typically involve the lower cervical or upper thoracic processes and are common in activities requiring rapid spinal rotation (Posthuma de Boer et al., 2016). Similarly, congenital variants—such as hypoplasia, dysplasia, or persistent apophyses—may mimic fractures and lead to misinterpretation if not correctly recognized (Riew et al., 2010; Farooqi et al., 2017; Gould et al., 2020). Understanding these variants is vital for distinguishing developmental anomalies from acute pathology and for avoiding unnecessary interventions (Chen et al., 2006). Beyond diagnosis, spinous process delineation also assists in evaluating outcomes following a spinal surgery called anterior cervical discectomy and fusion (ACDF), which is a widely performed procedure for degenerative conditions of the cervical spine. Postoperative evaluation of fusion status, indicating successful bony union and mechanical stability between adjacent vertebrae, represents a critical clinical outcome. However, studies show that inter-observer variability in assessing postoperative fusion from dynamic radiographs can be substantial, often requiring supplemental computed tomography (CT) scans or quantitative methods (Ariyaratne et al., 2024). With ACDF case volumes projected to rise markedly through 2040 (Neifert et al., 2020), automated segmentation tools could streamline assessment and reduce dependence on manual measurements.

Semantic segmentation—the pixel-level delineation of structures within images—is central to computational imaging and biomedical analysis (Hollon et al., 2020; Ouyang et al., 2020). Manual segmentation remains the reference standard but is labor-intensive, time-consuming, and prone to inter-observer variability (Ma et al., 2024). Automated methods can dramatically reduce workload, improve consistency, and enable high-throughput image analysis (Wang et al., 2019). However, manual labeling of X-rays is particularly challenging due to overlapping anatomy, limited soft-tissue contrast, and the absence of public datasets for small bony structures such as spinous processes (Wang et al., 2019; Wang S. et al., 2021; Galbusera and Cina, 2024). While recent advances in deep learning have achieved outstanding results for CT and MRI segmentation (Ahmad et al., 2023; Muthukrishnan et al., 2024; Son et al., 2024; Yang et al., 2024), segmentation in plain radiographs remains relatively underexplored.

To date, there is no publicly available dataset containing cervical spine X-rays with corresponding pixel-level annotations of the spinous processes. Prior work suggests that certain vertebral levels, notably C1/C2 and C6/C7, are particularly difficult to identify using machine learning models (Shim et al., 2022; van Santbrink et al., 2025). Preliminary zero-shot experiments using transformer-based encoders such as SAM (Bucher et al., 2019) and conventional segmentation architectures like DeepLabV3+ produced unsatisfactory results. These findings underscore the need for a domain-specific dataset and a dedicated segmentation framework optimized for the cervical spine.

Recent literature highlights that effective medical image segmentation models must balance local boundary precision with global contextual awareness. Conventional convolutional neural networks (CNNs) excel at fine detail but are limited in modeling long-range dependencies, whereas transformer architectures capture global context at the expense of spatial granularity. Hybrid models that integrate these complementary strengths have demonstrated improved performance across biomedical imaging tasks. Approaches such as MedSAM and TransUNet combine transformer-based encoders with U-Net–style decoders, yielding sharper boundaries and stronger structural consistency across diverse medical modalities (Chen J. et al., 2024; Ma et al., 2024). Similarly, hybrid convolution–transformer systems in musculoskeletal imaging achieve strong Dice and IoU performance and near-expert agreement (Xu et al., 2022; Ham et al., 2023; Mostafa et al., 2024; Al-Antari et al., 2025; Bao et al., 2025). These developments support hybrid encoder–decoder architectures as a robust design paradigm for accurate and data-efficient medical image segmentation.

Building on this foundation, the present study introduces CervSpineNet, a hybrid deep learning framework for automated segmentation of cervical spinous processes in lateral X-rays. The primary contributions are threefold:

1. A new expert-labeled dataset of cervical spine radiographs with pixel-level binary masks of the spinous processes.
2. A hybrid transformer–CNN architecture that integrates a ViT-B encoder for global context modeling with a traditional convolutional decoder with added layers for edge refinement.
3. A compound loss function that jointly optimizes region overlap, edge sharpness, class balance, and structural similarity.

CervSpineNet consistently achieved mean Dice coefficients exceeding 0.93 across experiments and demonstrated excellent agreement with ground-truth masks. Beyond accuracy, the framework reduces manual annotation time by approximately 96% and is efficient enough to run on standard hospital hardware. Together, the proposed dataset and model provide a scalable foundation for automated, high-fidelity cervical spine analysis, advancing both the methodological and translational frontiers of biomedical image segmentation.

## 2 Methodology

**FIGURE 1:**
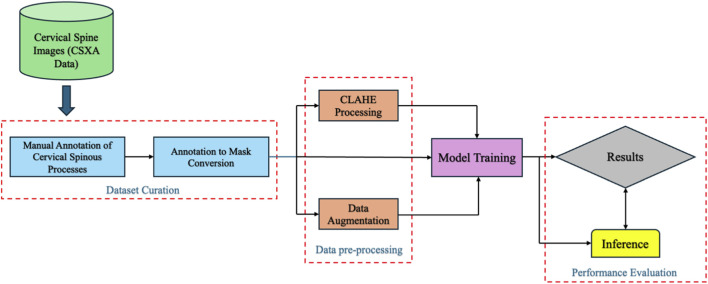
Schematic overview of the CervSpineNet pipeline for automated cervical spinous process segmentation. The workflow includes dataset curation from the publicly available Cervical Spine X-ray Atlas (CSXA), manual annotation of spinous processes, and annotation-to-mask generation for binary ground-truth creation. Preprocessing steps include Contrast Limited Adaptive Histogram Equalization (CLAHE) and data augmentation to enhance variability and image contrast. The processed datasets are used for model training and inference, with results evaluated through quantitative metrics and visual comparison to expert-annotated ground truth.

This section outlines the methodological framework used to develop and evaluate the proposed CervSpineNet model, from dataset curation and preprocessing to model training, benchmarking, and performance assessment. The overall workflow is illustrated in Figure 1.

### 2.1 Data acquisition

Prior work shows CNN-based segmentation is effective for vertebral bodies in radiographs and MRI (Chen Y. et al., 2024; Wang H. et al., 2025). However, no public dataset provides pixel-level masks of the cervical spinous processes, so we curated a task-specific corpus of lateral cervical spine X-rays and their corresponding binary masks.

**FIGURE 2:**
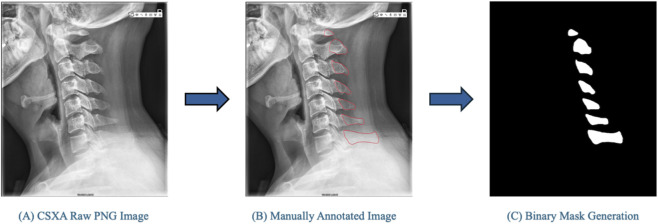
Representative workflow for creating binary masks of the cervical spinous processes from lateral X-ray images. **(A)** Original cervical spine radiograph from the Cervical Spine X-ray Atlas (CSXA) dataset. **(B)** Manual annotation of individual spinous processes using a tablet and stylus. **(C)** Automated mask conversion using a color-based segmentation algorithm in HSV space, where annotated red regions are thresholded and converted to binary masks. The resulting ground-truth masks serve as pixel-level labels for model training and evaluation.

We randomly sampled 500 PNG images from the Cervical Spine X-ray Atlas (CSXA) (Ran et al., 2024) (4,963 PNGs with JSON annotations; one image per patient) and manually annotated the spinous processes on a tablet with a stylus. Annotations were converted to binary masks using an OpenCV color-segmentation pipeline: images were converted to HSV; red hue bands (0 °–10 °, 160 °–180 °) corresponding to tracings were thresholded; masks from both bands were combined; contours were extracted and filled to produce clean binary ground-truth masks. All 500 image–mask pairs were created with consistent dimensions. Trained annotators labeled the images; an expert spine surgeon (Dr. Sangwook T. Yoon) provided final review and approval. The annotation workflow is shown in Figure 2.

### 2.2 Data bifurcation, pre-processing, and augmentation

To ensure fair evaluation and minimize overfitting (Muraina, 2022), we used an 80/20 split, allocating 400 images for training and 100 for testing (Bichri et al., 2024); the test set remained untouched throughout all experiments (Lones, 2021). For the training images, we generated three distinct dataset variants, each used to train a separate model configuration:

1. Original dataset: the 400 unaltered radiographs and their corresponding masks.
2. CLAHE dataset: the 400 original radiographs processed with Contrast Limited Adaptive Histogram Equalization (CLAHE) to compensate for non-uniform intensity distributions common in radiographs. Specifically, CLAHE operates by dividing the image into small contextual regions, equalizing each region’s histogram, and applying a clip limit to avoid noise amplification before recombining the tiles via bilinear interpolation (Zuiderveld, 1994). We used the standard OpenCV implementation.
3. Augmented dataset: the 400 original + 400 augmented image–mask pairs (800 total). To increase the effective training set size and improve model generalization (Goceri, 2023), augmentations including affine transformations with rotations of ±10 ° and ±45 ° and translations of ±10 pixels along x and y axes were applied to the original dataset. The larger ±45 ° rotations were included intentionally, as several expert-provided sample radiographs contained substantial initial misalignment. Mask transformations used nearest-neighbor interpolation to preserve labels.

All three training variants (original, CLAHE, and augmented) were derived from the same underlying set of 400 cervical radiographs. These variants reflect different preprocessing pipelines applied to identical base images rather than independent datasets, thus enabling us to evaluate how raw, intensity-normalized, and synthetically diversified training distributions affected model performance.

### 2.3 Experimental setup

We trained and evaluated each model on the three dataset variants: Original (400 images); CLAHE (400 images); Augmented (800 images). The test set (n = 100) remained constant across experiments. We compared GPU vs. CPU training/inference for efficiency. All experiments used a single system with PyTorch and an NVIDIA H100 Tensor Core GPU; the uniform environment ensured consistent timing and memory profiles. The H100’s tensor cores and memory bandwidth supported large-batch training and stable convergence.

A test across five different images from the testing set show that GPU inference averaged 5–8 s/image; CPU inference on an Intel® Xeon® Gold 6,136 (2 × 12 cores) averaged 9–10 s/image. The proposed CervSpineNet is computationally lightweight (∼345 MB) and typically utilizes ∼8–9 GPU cores during inference, enabling deployment without specialized hardware.

### 2.4 Proposed hybrid segmentation approach: CervSpineNet architecture

**FIGURE 3:**
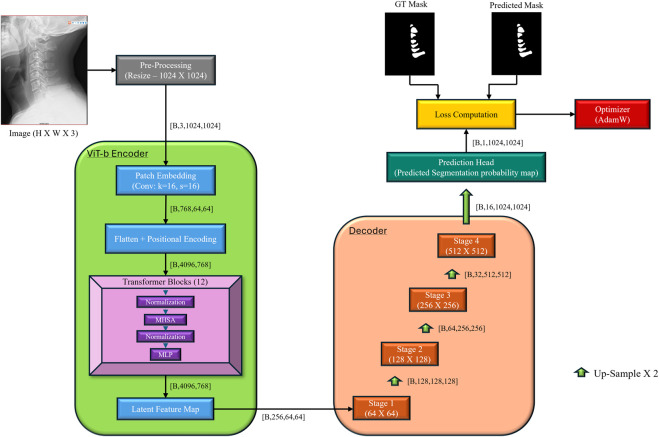
Schematic representation of the CervSpineNet hybrid architecture for automated segmentation of cervical spinous processes. The framework performs training and inference using paired cervical spine X-ray images and binary masks. CervSpineNet integrates a Vision Transformer (ViT-B) encoder for global contextual feature extraction with a U-Net–style decoder for spatial detail recovery. Each stage of the decoder includes residual blocks (RB) to maintain gradient flow, squeeze-and-excitation blocks (SEB) for adaptive channel attention, and bilinear upsampling (BU) layers for smooth boundary reconstruction. Preprocessing involves image resizing and binarization, while the output mask is generated via sigmoid activation and optimized using the composite loss function combining Dice, Focal Tversky, Hausdorff Distance Transform, and SSIM terms.

Figure 3 shows the overall architecture and I/O. The encoder is transformer-based; the decoder is a lightweight U-Net–style module with residual and squeeze-and-excitation (SE) blocks and bilinear up-sampling.

#### 2.4.1 Inputs and notations

We denote the input image by $x\in R^{3\times H\times W}$ and the binary target mask by $y\in {\left\{0,1\right\}}^{\left(1\times H\times W\right)}$ . Feature maps $F^{\left(\cdot \right)}$ with shape $\left(C\times h\times w\right).$ Convolutions use kernel K and bias b . Moreover, * is convolution and $\sigma \left(\cdot \right)$ is sigmoid.

#### 2.4.2 Encoder: ViT-B

Vision transformers (ViT) capture global context and long-range dependencies valuable for medical segmentation (Zhang et al., 2024), but benefit from local refinements from downstream decoders (Khan and Khan, 2025). As a result, we base our encoder on the ViT-B backbone of the Segment Anything Model (SAM). Specifically, we load the ViT-B variant from the original SAM repository through the sam_model_registry interface using the official PyTorch implementation of SAM. The prompt encoder and mask decoder heads are eliminated from this model, leaving only the image encoder.

Given an input radiograph/x-ray image that is resized to 1,024×1,024 and rescaled to [0,1], the SAM ViT-B image encoder processes the radiograph and yields a dense feature map $F^{enc}$ with 256 channels at a spatial resolution of 64×64 as the output. This is given by Equation 1.

$$F^{enc}={ViT}_{SAM}\left(x\right)\in R^{256\times 64\times 64}$$ (1)

Our unique U-Net-style decoder receives these feature maps and uses multi-scale up sampling to return the image to its original resolution.

The encoder is based on the SAM ViT-B image encoder, which we use as a generic feature extractor for cervical spine radiographs. Each input radiograph is first resized to 1,024 × 1,024 and linearly projected into a sequence of non-overlapping 16 × 16 patches. These patches are embedded into a high-dimensional token space, augmented with learned 2D positional encodings, and passed through a stack of multi-head self-attention and feedforward transformer blocks. Across these layers, the encoder aggregates information over the entire field-of-view, so that each token encodes both local appearance (e.g., vertebral boundaries) and long-range context (e.g., overall spinal alignment). Finally, the token sequence is reshaped back into a 2D feature map of size 64 × 64 with 256 channels, which serves as the latent representation fed into our U-Net–style decoder for pixel-wise mask prediction.

The encoder weights are initialized from the SA-1B pre-trained SAM ViT-B checkpoint and then fine-tuned end-to-end together with our decoder on the curated cervical spine dataset using the compound loss described in Section 2.4.4. In contrast, we also train a complete SAM baseline (Section 2.5.3) that keeps the original image encoder, prompt encoder, and mask decoder. In this baseline, the entire SAM model is optimized using the same training set, and centroids obtained from the ground-truth masks are utilized as point prompts.

To qualitatively consider if the ViT-B encoder in CervSpineNet uses global contextual information, we generated three types of complementary attention maps for the trained hybrid model. During a forward pass, we cached the self-attention inputs to all transformer blocks and the final encoder feature map. First, we computed an attention-rollout map by creating the self-attention matrix for each block at the final token resolution, adding an identity term, normalizing rows, and multiplying the matrices across blocks; the vector of token importances that resulted was reshaped to the encoder grid (≈64 × 64). Second, we computed a query-centric attention map from the last block by selecting a query token centered on the mid-cervical spinous process and visualizing its attention weights to all other tokens. Third, we produced a Grad-CAM map on the output of the encoder by back-propagating the mean foreground prediction to the encoder feature map and aggregating gradient-weighted activations. All maps were upsampled to image resolution and overlaid with the radiograph for visual interpretation.

#### 2.4.3 Decoder

U-Net decoders progressively restore spatial resolution and boundary fidelity (Yuan and Cheng, 2024). Our decoder stages include Conv + ReLU → Residual Block → SE Block → Bilinear Upsample → 1 × 1 Conv + Sigmoid. SE blocks re-weight channels to enhance task-relevant features (Wang J. et al., 2021); residual blocks maintain gradient flow and stabilize training (Ashkani Chenarlogh et al., 2022).

##### 2.4.3.1 Convolution stage

$$F_{O}=\phi \left(K*F+b\right)$$ (2)

Equation 2 denotes the convolution stage where F is the input feature map and $F_{O}$ is the resulting output feature map; * denotes convolution, $K\in R^{C_{out}\times C_{in}\times 3\times 3}$ is the convolution kernel, b is the bias term, and ϕ is the ReLU activation function.

##### 2.4.3.2 Residual block

$$ResBlock\left(F\right)=F+\phi \left(K2*\phi \left(K1*F+b1\right)+b2\right)$$ (3)

The residual block depicted by Equation 3 contains two stacked Conv + ReLU layers with a skip connection. K1 and K2 are the first and second convolution layers, and b1 and b2 are the bias terms for each respective layer.

##### 2.4.3.3 SE block—squeeze (global average pooling)

$$z_{c}=\frac{1}{hw}\sum_{i,j}F_{c,i,j}$$ (4)

Equation 4 gives the squeeze formula for the SE block where $F_{c,i,j}$ is the activation value at channel c and spatial location $\left(i,j\right)$ , h and w are the height and width of the feature map, and $z_{c}$ is the global descriptor for channel c . This step compresses spatial information into a single number per channel.

##### 2.4.3.4 SE block—excitation (2-layer MLP)

$$s=\sigma \left(W2\phi \left(W1z\right)\right)$$ (5)

Equation 5 gives the excitation formula for the SE block where s is the vector of channel weights, z is the vector of all $z_{c}$ values, W1 is the first and W2 is the second fully connected layer, and W1z denotes the linear transformation applied to the channel descriptor z . σ and ϕ are the Sigmoid and ReLU activations respectively.

##### 2.4.3.5 Channel re-weighting

$$SE{\left(F\right)}_{c,i,j}=s_{c}\times F_{c,i,j}$$ (6)

Channel re-weighing is represented by Equation 6, where $s_{c}$ is the weight from the excitation step, and × denotes element-wise multiplication with broadcasting of $s_{c}$ across all spatial locations $\left(i,j\right)$ in channel c .

##### 2.4.3.6 Bilinear upsampling

$$F^{⊖}={Upsample}_{\times 2}\left(F\right)\in R^{C\times \left(2h\right)\times \left(2w\right)}$$ (7)

The output feature map *F^⊖^* after sampling is given by Equation 7. F and C are the input feature map and number of channels respectively as mentioned earlier, and ${Upsample}_{\times 2}\left(F\right)$ is bilinear interpolation. Each stage of the decoder contains one ${Upsample}_{\times 2}\left(F\right)$ operation. These operations increase the spatial resolution of the feature map F by a factor of two in both height and width using bilinear interpolation.

Our decoder is U-Net–inspired but intentionally departs from the textbook U-Net design. Because the encoder is a SAM ViT-B transformer that produces a single low-resolution feature map (256 channels at 64 × 64), the decoder operates on this representation only and does not use multi-scale encoder–decoder skip connections or a symmetric convolutional contracting path. Instead, it forms a purely expanding path with four stages of bilinear up-sampling followed by 3 × 3 convolutions, residual blocks, and SE blocks that refine features at each scale before the final 1 × 1 convolution and sigmoid.

#### 2.4.4 Loss function

Composite losses can improve robustness by balancing region overlap, boundary accuracy, and imbalance (Taghanaki et al., 2019; Mu et al., 2022). To jointly optimize region-wise overlap, class imbalance, boundary accuracy, and structural fidelity, we selected the four loss elements: Dice, Focal Tversky (FT), Hausdorff Distance Transform (HD95), and Structural Similarity Index (SSIM). The Hausdorff Distance Transform term specifically penalizes boundary misalignment, the SSIM term promotes structurally coherent, non-blurry masks that respect the overall spine morphology, Focal Tversky down-weights easy background pixels and concentrates learning on imbalanced, thin vertebral structures, and Dice loss offers a powerful region-overlap term.

Let $\hat{y}\in {\left[0,1\right]}^{\Omega }$ be the predicted probability map and $y\in {\left\{0,1\right\}}^{\Omega }$ be the ground-truth mask over pixel set Ω with $\left|\Omega \right|=N$ , and a small constant ε for numerical stability.

The Dice Loss is given by Equation 8:

$${Loss}_{dice}\left(\hat{y},y\right)=1-\frac{2\sum_{i\in \Omega }{\hat{y}}_{i\;}y_{i}+\varepsilon }{\sum_{i\in \Omega }{\hat{y}}_{i\;}+\sum_{i\in \Omega }y_{i}+\varepsilon }$$ (8)

For the Focal Tversky loss, we first compute the True Positive (TP), False Positive (FP) and False Negative (FN) as given in Equation 9:

$$TP=\sum_{i\in \Omega }{\hat{y}}_{i}y_{i},FP=\sum_{i\in \Omega }{\hat{y}}_{i}\left(1-y_{i}\right),FN=\sum_{i\in \Omega }\left(1-{\hat{y}}_{i}\right)y_{i}$$ (9)

Thus, the Tversky Index, represented by Equation 10, is given by

$$T\left(\hat{y},y\right)=\frac{TP+\varepsilon }{TP+\alpha FN+\beta FP+\varepsilon }$$ (10)

And the Focal Tversky Loss, given by Equation 11, is computed as:

$${Loss}_{FocalTversky}\left(\hat{y},y\right)={\left(1-T\left(\hat{y},y\right)\right)}^{\gamma }$$ (11)

Where, α=0.75,β=0.3,and γ=0.75 in our experiments.

For the Hausdorff Distance Transform Loss, depicted by Equation 12, we form binarized masks, $\overset{˘}{y}=1\left[\hat{y}>0.5\right]$ and $y_{i}$ , and compute their Euclidean distance transforms $D_{\overset{˘}{y}\;}$ and $D_{y}$ . For each pixel i , $D_{\overset{˘}{y},i\;}$ is the distance to the nearest boundary of $\overset{˘}{y}$ , and similarly for $D_{y,i}$ . The loss averages the absolute difference between these distance fields:

$$\left.{Loss}_{HausdorffDT}\left(\hat{y},y\right)=\frac{1}{N}\;\left.\sum_{i\in \Omega }\right|D_{\overset{˘}{y},i\;}-D_{y,i}\right|$$ (12)

Finally, for the SSIM loss given by Equation 13, we compute the image-wise average SSIM following the standard definition (Equation 18), and define the loss as:

$${Loss}_{SSIM}\left(\hat{y},y\right)=1-SSIM\left(\hat{y},y\right)$$ (13)

After ablations with the composition of loss functions (Section 2.7.2), the best results were obtained with a weighted sum of Dice, Focal Tversky, Hausdorff Distance Transform, and SSIM (Equation 14):

$${Loss}_{Total}=\left[0.5\times {Loss}_{Dice}\left(\hat{y},y\right)\right]+\left[0.3\times {Loss}_{FocalTversky}\left(\hat{y},y\right)\right]+\left[0.1\times {Loss}_{HausdorffDT}\left(\hat{y},y\right)\right]+\left[0.1\times {Loss}_{SSIM}\left(\hat{y},y\right)\right]$$ (14)

Based on our pilot experiments, we empirically selected the weights (0.5, 0.3, 0.1, 0.1). We prioritize Dice as the primary region-overlap term, give Focal Tversky a moderate weight to address class imbalance, and use smaller regularization weights for the boundary-focused HDT and SSIM terms to sharpen contours without destabilizing optimization.

### 2.5 Baselines implemented: performance comparison

To avoid model-class bias and follow current benchmarking guidance (Antonelli et al., 2022; Tejani et al., 2024), we compared CervSpineNet to strong and architecturally diverse baselines (Wolfrath et al., 2024): DeepLabV3+, U-Net, the full Segment Anything Model (SAM), and a text-guided SegFormer. All baselines and CervSpineNet were trained and evaluated on the same training/test split with identical pre-processing and data augmentations. U-Net, DeepLabV3+, and text-guided SegFormer were trained on images resized to 512 × 512, which is standard for CNN-based medical segmentation and keeps GPU memory requirements manageable. The SAM ViT-B and the proposed CervSpineNet were trained on 1,024 × 1,024 inputs to match the native input resolution of the SAM image encoder. All predictions were resampled back to the original image resolution for evaluation. The batch size for every training experiment was kept 1, and the models were trained on 50 epochs, selecting the checkpoint with the best testing metrics as the metrics saturated around 40–45 for all experiments. This ensured that the performance differences mainly reflect architectural choices rather than differences in data or optimization.

#### 2.5.1 DeepLabV3 +

DeepLabV3+ performs strongly across medical segmentation tasks (Hou et al., 2024; Ketenci Çay et al., 2025). We implemented DeepLabV3+ with a ResNet-50 backbone pretrained on ImageNet. The single-channel radiographs were resized to 512 × 512 pixels and duplicated to three channels. A 1-channel logit map that has been upsampled to the input resolution is produced by the model. An AdamW optimizer and BCE Loss were used to refine DeepLabV3+ on our cervical dataset.

#### 2.5.2 U-net

U-Net remains a robust baseline across modalities (Ronneberger et al., 2015; Du et al., 2020; Azad et al., 2022). We created a 4-level U-Net from scratch using 64 initial filters as a convolutional baseline. The inputs were 512 × 512. We used the AdamW optimizer with BCE Loss.

#### 2.5.3 Segment anything model (SAM)

SAM is trained on >1B masks (Kirillov et al., 2023) and, with task-specific prompting, can be adapted to medical images (Mazurowski et al., 2023). For SAM, we used the official ViT-B variant from the Meta Segment-Anything repository, loaded via the sam_model_registry. We kept the full architecture, namely image encoder, prompt encoder, mask decoder, and resized X-rays to 1,024 × 1,024 as recommended. For each training image, we derived oracle point prompts by placing a single positive point at the centroid of each ground-truth spinous process mask. We passed these points through the prompt encoder and supervised the resulting masks with binary cross-entropy + Dice loss. We then fine-tuned all SAM parameters with the AdamW optimizer for 50 epochs. At inference time, we used one positive centroid point per spinous process and placed a threshold for the output probabilities at 0.5. This setup follows recent SAM adaptations to medical imaging and represents a reasonable, strong SAM baseline for our task.

#### 2.5.4 Vision-language model—text guided SegFormer

We used MedCLIP (domain-adapted CLIP) (Wang et al., 2022) to encode text prompts describing the spinal processes; embeddings were L_2_-normalized and pooled to a fixed vector $t\in R^{dt}$ . SegFormer employs a hierarchical MiT encoder and lightweight MLP. We modulated the final encoder stage with the text embedding via a linear projection and channel-wise scaling; the decoder produced logits upsampled to input resolution and passed through sigmoid. For text guidance, we created brief, anatomy-focused prompts for every target structure and used MedCLIP to encode them once. Only the SegFormer parameters were optimized on our training set, and the text encoder was kept frozen. For the decoder to predict masks that were explicitly conditioned on the selected prompt, the resulting text embedding was broadcast to every pixel and used to modulate the final MiT feature map via channel-wise scaling. This configuration replicates current text-guided segmentation baselines and gives our hybrid model a fair, repeatable benchmark without requiring further task-specific language backbone tuning.

### 2.6 Evaluation metrics

Following common practice (Li et al., 2025; Zhang et al., 2025), we report Dice, Intersection-over-Union (IoU), Structural Similarity (SSIM), Hausdorff Distance (HD95), and Volumetric Similarity (VS) on the held-out test set. Equations 15-17 display the Dice, IoU and VS terms as implemented in this study.

$$Dice=\frac{2\cdot TP}{2\cdot TP+FP+FN}$$ (15)

$$IoU=\frac{TP}{TP+FP+FN}$$ (16)

$$VS=1-\frac{\left|\left(TP+FP\right)-\left(TP+FN\right)\right|}{\left|\left(TP+FP\right)+\left(TP+FN\right)\right|}$$ (17)

Here, TP/FP/TN/FN are computed from thresholded (0.5) binary predictions and ground-truth masks for each pixel.

- True Positive (TP) – prediction = 1, ground truth = 1 (pixels correctly classified as foreground)
- False Positive (FP) – prediction = 1, ground truth = 0 (pixels incorrectly classified as foreground)
- True Negative – prediction = 0, ground truth = 0 (pixels correctly classified as background)
- False Negative (FN) – prediction = 0, ground truth = 1 (missed foreground pixels)

SSIM captures luminance, contrast, and structure similarity. We used the standard SSIM metric (Bakurov et al., 2022), defined for a predicted mask x and ground truth mask y as shown in Equation 18:

$$SSIM\left(x,y\right)=\frac{\left(2\mu_{x}\mu_{y}+C1\right)\left(2\sigma_{xy}+C2\right)}{\left(\mu_{x}^{2}+\mu_{y}^{2}+C1\right)\left(\sigma_{x}^{2}+\sigma_{y}^{2}+C2\right)}$$ (18)

where, $\mu_{x}{\;and\;\mu }_{y}$ are the mean pixel intensities of x and y , $\sigma_{x}^{2}\;and\;\sigma_{y}^{2}$ are the corresponding variances, $\sigma_{xy}$ is the cross-covariance between x and y ; C1,C2 are small constants for stability.

HD95, as given by Equation 20, is the 95th percentile surface-to-surface distance between predicted and ground-truth objects (Ramachandran et al., 2023). Let A,B be sets of foreground boundary points with Euclidean distance *d*. The directed surface distance sets are calculated as in Equation 19:

$$D\left(A,B\right)=\left\{\min_{b\in B}\;d\left(a,b\right)|a\in A\right\},D\left(B,A\right)=\left\{\min_{a\in A}\;d\left(b,a\right)|b\in B\right\}$$ (19)

Thus, for all distances, $S=D\left(A,B\right)\cup D\left(B,A\right)$ .

$$HD95\left(A,B\right)={percentile}_{95}\left(S\right)$$ (20)

### 2.7 Ablation studies

Ablation experiments are essential in medical image segmentation, particularly for hybrid architectures, as they clarify the relative contributions of individual components to overall performance (Gao et al., 2021; Wang X. et al., 2025). To systematically characterize the behavior of CervSpineNet, we performed two sets of ablations: (i) architectural ablations isolating encoder and decoder contributions, and (ii) loss-function ablations assessing how each loss term influences error modes and structural fidelity.

#### 2.7.1 Architectural ablation experiments

To quantify how much performance arises from the encoder versus the decoder, we evaluated three architectural variants under identical training conditions, preprocessing, composite loss, and data splits.

- Pure CNN Encoder–Decoder Baseline

We first constructed a fully convolutional baseline in which both the encoder and decoder are simple CNNs. The encoder comprises four convolution–BatchNorm–ReLU blocks, each followed by 2 × 2 max pooling, reducing the spatial resolution from 1,024 × 1,024 to 64 × 64 while expanding feature channels from 3 to 256. The decoder is deliberately minimal, consisting of stacked 3 × 3 convolutions with ReLU activations and bilinear upsampling, terminating in a 1 × 1 sigmoid layer for mask generation. No residual connections, attention modules, or skip connections are used. This configuration provides a reference model that isolates the performance of a straightforward fully convolutional architecture.

ii. CNN Encoder + Full Decoder

Next, we evaluated a model with the same CNN encoder as above but augmented with our full decoder. Each decoding stage includes a 3 × 3 convolution, a residual block to enhance representational depth without compromising gradient flow, and a squeeze-and-excitation (SE) block for adaptive channel reweighting based on global context. Bilinear upsampling is applied at each stage. Because the encoder and training protocol remain constant, differences in performance directly reflect the contribution of residual pathways and channel attention to anatomical structure reconstruction.

iii. ViT-B Encoder (SAM) + Simple Decoder

Finally, we substituted the CNN encoder with the Vision Transformer–B (ViT-B) image encoder from Segment Anything, while retaining the simple decoder of the first experiment. The ViT-B encoder processes 1,024 × 1,024 inputs to produce 256-channel 64 × 64 feature maps enriched with long-range dependencies via self-attention. All transformer layers are unfrozen during training. Using the same composite loss, optimizer (AdamW), and data splits allows this ablation to cleanly assess the effect of replacing local convolutional features with global transformer-based representations, independent of decoder capacity.

#### 2.7.2 Loss ablation experiments

We also performed a dedicated loss-function ablation using the full CervSpineNet architecture (ViT-B encoder + residual/SE decoder). The optimizer, learning rate schedule, and data partitions were held constant across conditions. Loss terms were added progressively to address complementary error modes.

Beginning with a Dice-only baseline emphasizing volumetric overlap, we incorporated the Focal Tversky (FT) loss to penalize false negatives—critical for foreground–background imbalance in postoperative cervical spine X-rays. We then added the SSIM term to encourage structural and textural fidelity, particularly along vertebral margins and disc spaces. Finally, we introduced a differentiable Hausdorff distance surrogate (HD95) to explicitly penalize boundary deviations and outlier predictions.

This yielded five configurations—Dice; Dice + FT; Dice + SSIM; Dice + FT + SSIM; and Dice + FT + SSIM + HD95—each trained for 50 epochs and evaluated on an identical test set using Dice, IoU, SSIM, HD95, and volumetric similarity metrics.

## 3 Results

This section presents the outcomes of the experiments designed to evaluate the proposed CervSpineNet model and its comparative baselines. Quantitative results are reported across the original, CLAHE-enhanced, and augmented datasets, followed by statistical significance testing, qualitative visual assessments, and an analysis of time efficiency between automated and manual segmentation.

### 3.1 Quantitative comparison

Mean performance metrics (Dice, IoU, SSIM, HD95, VS) on the testing data were computed for all models across the three dataset variants: the original set (Table 1), CLAHE-enhanced images (Table 2), and the augmented dataset (Table 3).

**TABLE 1:** Quantitative evaluation of segmentation models on the original dataset.

Models	Dice	IoU	SSIM	HD95	VS
SAM	0.8553	0.7532	0.9752	25.744	0.9132
U-net	0.9013	0.8226	0.969	**3.1296**	0.9726
Text-guided SegFormer	0.9266	0.8641	0.9778	4.2251	0.9819
DeepLabV3+	0.9287	0.8676	0.9781	4.0418	**0.9831**
CervSpineNet	**0.9315**	**0.8726**	**0.9831**	3.3549	0.9818

Mean Dice, IoU, SSIM, HD95, and Volumetric Similarity (VS) scores are reported on the held-out test set (n = 100). CervSpineNet achieved the highest Dice and SSIM, indicating strong overlap and structural fidelity, while maintaining the lowest HD95 (better boundary accuracy).

Across all tables, numeric values in bold font indicate the best mean score yielded

**TABLE 2:** Quantitative evaluation on the CLAHE-enhanced dataset.

Models	Dice	IoU	SSIM	HD95	VS
SAM	0.8246	0.7103	0.9733	27.555	0.8929
U-net	0.9033	0.8268	0.9707	3.4191	0.9598
Text-guided SegFormer	0.9250	0.8614	0.9776	4.2898	0.9778
DeepLabV3+	0.9260	0.8631	0.9778	4.7765	**0.9806**
CervSpineNet	**0.9313**	**0.8722**	**0.9829**	**2.6561**	0.9777

Performance comparison of five segmentation models on contrast-normalized images. CervSpineNet maintained consistent superiority across Dice, SSIM, and HD95, demonstrating robustness to illumination and contrast variations typical of radiographs.

Across all tables, numeric values in bold font indicate the best mean score yielded

**TABLE 3:** Quantitative evaluation on the augmented dataset.

Models	Dice	IoU	SSIM	HD95	VS
SAM	0.7955	0.6698	0.9722	28.8584	0.8435
U-net	0.9099	0.8367	0.9712	3.0973	0.9721
Text-guided SegFormer	0.9289	0.8679	0.9781	4.0540	0.9820
DeepLabV3+	0.9303	0.8704	0.9784	3.7779	**0.9838**
CervSpineNet	**0.9326**	**0.8744**	**0.9832**	**2.3806**	0.9833

Results of all segmentation models after data augmentation with rotations and translations. CervSpineNet again produced the best or near-best mean scores across all metrics, confirming generalization under data diversity.

Across all tables, numeric values in bold font indicate the best mean score yielded

**FIGURE 4:**
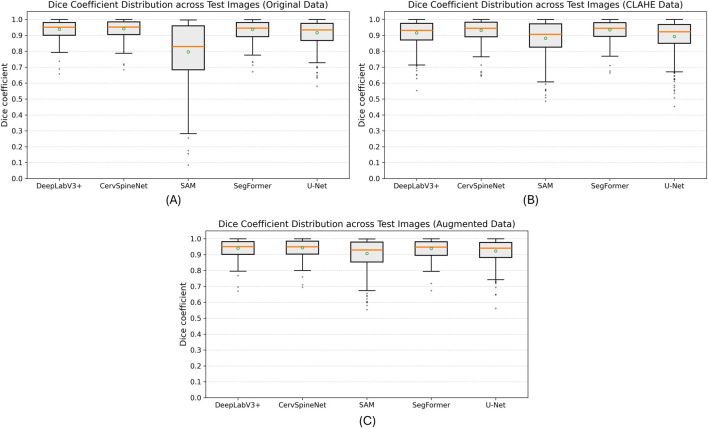
Distribution of Dice coefficients across test datasets for different preprocessing conditions. Boxplots show per-image Dice scores (n = 100) for all segmentation models evaluated on **(A)** the original dataset, **(B)** the CLAHE-enhanced dataset, and **(C)** the augmented dataset. CervSpineNet demonstrates the highest median Dice and the smallest interquartile range across all three data variants, indicating superior segmentation accuracy and stability relative to baseline models.

Repeated trials with identical hyperparameters produced standard deviations in the range 0.001–0.005 for Dice, IoU, SSIM, and VS and ∼0.3–0.6 for HD95, confirming the stability and reproducibility of model performance across the testing data. Figure 4 shows the distribution of Dice coefficients for all models evaluated on the 100 test images under the three preprocessing conditions: (A) original data, (B) CLAHE-enhanced data, and (C) augmented data. Across all three dataset variants, CervSpineNet exhibited the narrowest spread and highest median Dice scores, indicating robustness to preprocessing variations and the radiographic variability that these variants are designed to reflect.

CervSpineNet achieved the highest or near-highest mean values for nearly every metric, with particularly strong performance in SSIM and HD95, which reflect structural accuracy and boundary precision, respectively. To determine whether these improvements were statistically significant, formal non-parametric tests were conducted as described in the following section.

A summary of the mean test-set performance for all four architectural configurations adopted in this study is shown in Table 4.

**TABLE 4:** Architectural Ablation Experiments and the metrics yielded: This table follows a similar structure as Tables 1–3 and demonstrates a comparison between different ablation experiments done with the hybrid model architecture utilizing the same metrics as used previously.

Experiment	Dice	IoU	SSIM	HD95	VS
Pure CNN + basic decoder	0.8516	0.7616	0.9828	33.9749	0.9222
Pure CNN + full decoder	0.8852	0.8021	0.9849	20.175	0.9534
ViT-b encoder + basic decoder	0.9307	0.8713	**0.9886**	8.8335	0.9804
ViT-b encoder + full decoder (CervSpineNet)	**0.9315**	**0.8726**	0.9831	**3.3549**	**0.9818**

Across all tables, numeric values in bold font indicate the best mean score yielded

Finally, the results of the loss ablation experiment are demonstrated in Table 5. Different sets of loss functions used, and their metrics scores are depicted in this table. The experiments demonstrated in Tables 4, 5 also yielded minute variations in the range of 0.002–0.004 for Dice, IoU, SSIM, and VS and variations of ∼0.45–0.70 for HD95.

**TABLE 5:** Loss Ablation Experiments and the metrics yielded: different combinations of loss functions and their results.

Experiment	Dice	IoU	SSIM	HD95	VS
Dice	0.9153	0.8457	0.9815	5.8591	0.9694
Dice + FT	0.9288	0.868	0.9826	3.9504	0.9743
Dice + SSIM	0.931	0.8719	0.9832	3.8787	0.9812
Dice + FT + SSIM	0.9286	0.868	0.9829	5.0694	0.979
Dice + FT + SSIM + HD95 (CervSpineNet)	**0.9315**	**0.8726**	**0.9831**	**3.3549**	**0.9818**

FT indicates Focal Tversky Loss; SSIM is Structural Similarity Index Loss; HD95 represents 95th percentile of Hausdorff Distance Loss.

Across all tables, numeric values in bold font indicate the best mean score yielded

### 3.2 Statistical significance analysis of model performances

Prior studies highlight the robustness of the Wilcoxon Signed-Rank test and the Friedman test, which detects overall performance differences across multiple models evaluated on the same samples, for evaluating segmentation algorithms (Mostafa et al., 2024). We compared five segmentation models (SAM, U-Net, text-guided SegFormer, DeepLabV3+, and CervSpineNet) using five metrics (Dice, IoU, SSIM, HD95, and VS) across the same per-image testing scores.

The Friedman test assessed overall model differences for each metric, followed by pairwise Wilcoxon signed-rank tests with Bonferroni correction (p < 0.05). Across all metrics, significant overall differences were observed (Friedman p < 0.001).

CervSpineNet significantly outperformed SAM and U-Net (corrected p < 0.001) in every metric and dataset variant. It also exceeded DeepLabV3+ in SSIM, HD95, and Volumetric Similarity while maintaining comparable Dice and IoU (corrected p ≈ 0.05). Against the text-guided SegFormer, CervSpineNet achieved statistically higher performance in all metrics except VS, where the difference was not significant. These results confirm that the proposed hybrid model, CervSpineNet, provides robust and consistent segmentation.

### 3.3 Qualitative inference visualization and error analysis

**FIGURE 5:**
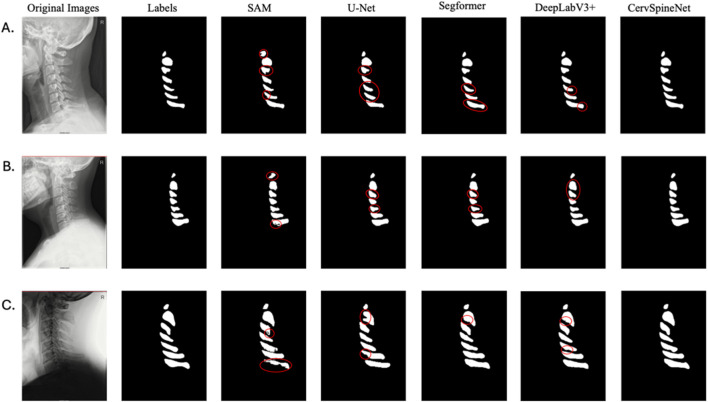
Qualitative comparison of model predictions on representative test radiographs. Representative examples **(A–C)** show three randomly selected test radiographs and their corresponding segmentation results for each baseline model and the proposed CervSpineNet. As evidenced by the red markings, CervSpineNet demonstrates superior boundary sharpness and anatomical conformity of the spinous processes compared with U-Net, DeepLabV3+, SAM, and the text-guided SegFormer. The red circles display the visual prediction errors made by the baselines when compared to the ground-truth mask.

Visual inspection of unseen test radiographs further corroborated the quantitative findings (Figure 5). Panels **(A)**, **(B)**, and **(C)** show representative segmentation results from the original, CLAHE-enhanced, and augmented datasets, respectively, comparing **CervSpineNet** with all baseline models. Across all preprocessing conditions, **CervSpineNet** produced the most accurate and anatomically consistent delineations, with smoother contours, sharper boundaries, and markedly fewer false or incomplete predictions than either CNN-only or transformer-only architectures. These qualitative outcomes parallel the quantitative improvements reported in Section 3.1, illustrating how the hybrid architecture captures global contextual features while preserving fine boundary details.

**FIGURE 6:**
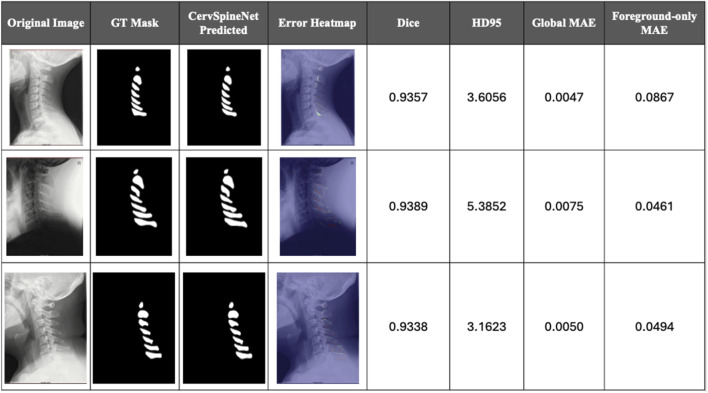
Qualitative error analysis and per-image Dice, HD95, and mean absolute error (MAE) for high-quality CervSpineNet predictions. Representative examples show original cervical spine X-rays, expert ground-truth (GT) masks, CervSpineNet-predicted masks, and corresponding error heatmaps. Colored overlays highlight mis-segmented regions, where greater intensity denotes larger deviation from the GT boundaries. Quantitative values include Dice, HD95, Global MAE (overall pixel-wise deviation) and Foreground-only MAE (error within annotated regions). Lower values indicate higher structural fidelity and more accurate boundary reconstruction.

To examine segmentation fidelity in more detail, Figure 6 visualizes per-pixel discrepancies between CervSpineNet predictions and expert ground-truth masks. In these heatmaps, uncolored regions indicate perfect agreement, while increasing color intensity reflects greater deviation from the annotated boundaries. False positives represent regions erroneously predicted as foreground outside the annotated structure, false negatives correspond to missed spinous process pixels within the ground-truth region, and thin color bands along the contours indicate minor boundary mismatches or thickness differences.

**FIGURE 7:**
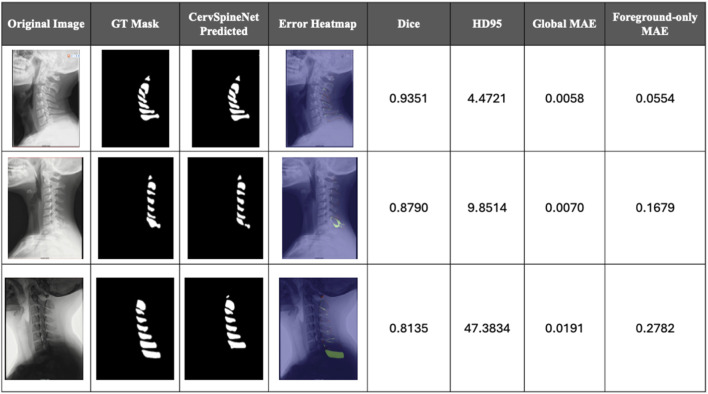
Qualitative error analysis and per-image Dice, HD95, and mean absolute error (MAE) for low-quality CervSpineNet predictions. It is like Figure 6 in terms of the results displayed but this figure demonstrates the failure modes of the hybrid model and includes some edge case radiographs that causes the model to predict masks poorly and yield below-par metric results.

A complementary quantitative error assessment was performed using Dice, HD95, and Mean Absolute Error (MAE) calculated on continuous (non-binarized) probability maps to preserve boundary sensitivity. Figure 6 demonstrate a sample of radiographs with good model predictions backed by the metrics used for comparison. On the other hand, Figure 7 depicts some edge cases where the model failed to perform well due to a variety of reasons often resulting in over/under prediction of structures or significant disagreement from ground truth masks.

A pixel-by-pixel error heatmap between the ground-truth spinous process mask and the model prediction is superimposed on the lateral cervical X-ray in Figures 6, 7. Areas where prediction and ground truth agree appear like the original image, whereas colored bands around the spinous processes mark disagreement: cooler colors indicate small or no error, and warmer colors (green–yellow–red) highlight increasing mismatch, with the brightest regions corresponding to the largest segmentation errors. In these figures, the Global MAE captures the average pixel-wise deviation across the entire image, reflecting overall segmentation fidelity, whereas the Foreground-only MAE quantifies deviation exclusively within annotated spinous process regions, emphasizing local structural precision. Both metrics were computed at the native image resolution, where lower MAE values indicate stronger correspondence with expert annotations. CervSpineNet consistently achieved the lowest Global and Foreground-only MAE scores, confirming high boundary accuracy and strong structural alignment across the test set.

Figure 6 shows three representative test radiographs with high-quality CervSpineNet segmentations. In these typical cases, spinous process masks from C1–C7 closely follow the cortical margins with minimal over- or under-segmentation. These examples support the good overall performance observed in the aggregate metrics by achieving high quantitative agreement with the reference annotations (Dice ≈ 0.93, HD95 ≈ 3, Global MAE ≈ 0.005, and Foreground-only MAE ≈ 0.05).

Figure 7 illustrates three characteristic failure modes encountered by CervSpineNet. In Case 1, adjacent spinous processes are extremely close and partially overlapping, causing the model to merge two levels (C3–C4) into a single elongated mask and consequently overestimate the superior spinous process. In Case 2, a fractured spinous process produces an irregular and discontinuous contour. While the expert ground truth precisely outlines the fracture margins, the model predicts a smoother, unbroken process, resulting in reduced Dice scores and a prominent error band in the heatmap. In Case 3, the C7 spinous process is scarcely visible due to low contrast and soft-tissue overlap at the cervicothoracic junction; although the model accurately segments C1–C6, it fails to detect C7 entirely, yielding a level-specific false negative. Together, these examples indicate that severe pathology, low bone–soft-tissue contrast, and tightly packed vertebral levels are the conditions under which CervSpineNet is most vulnerable. Future work will prioritize augmenting the training set with such challenging cases and exploring level-aware constraints or post-processing strategies to mitigate mask merging and missed levels.

**FIGURE 8:**
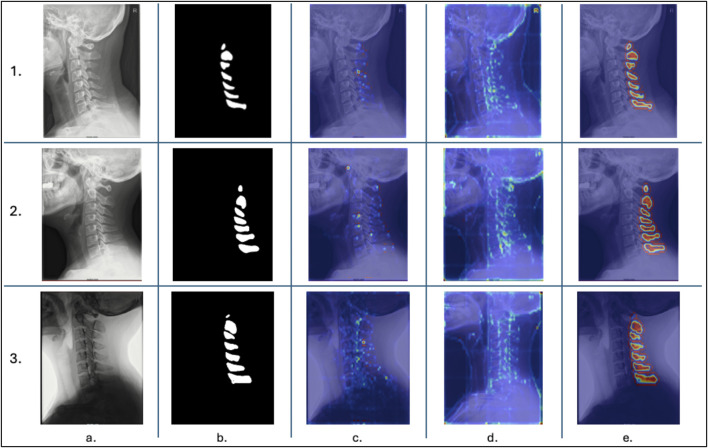
Transformer Attention Maps for ViT-B. Attention maps generated for three test cases; two with good (1, 2) and one with bad metrics and predicted masks (3). **(a)** is the original radiograph, **(b)** is the hybrid model predicted mask, **(c)** is the query centric map, **(d)** represents the attention-rollout map, and **(e)** depicts the Grad-CAM map.

Figure 8 presents attention visualizations for three representative held-out test cases—two typical high-quality segmentations and one challenging example with substantial shoulder overlap. In the well-segmented cases, both attention-rollout and Grad-CAM overlays highlight a continuous chain of vertebral bodies and spinous processes spanning the upper through lower cervical levels. This pattern indicates that the transformer encoder integrates information across the full cervical column rather than relying solely on local image patches. Similarly, the query-centric attention map, when the query token is placed on a mid-cervical spinous process, assigns high attention weights to both adjacent and more distant vertebral levels, reinforcing evidence of long-range contextual reasoning. In contrast, the difficult case shows more spatially diffuse attention that partially shifts toward the overlapping shoulder and soft-tissue regions, corresponding to the observed segmentation errors. Collectively, these qualitative results support the conclusion that CervSpineNet’s transformer encoder leverages global anatomical context along the cervical spine.

### 3.4 Comparison of manual and automated segmentation efficiency

We conducted a systematic analysis to compare the time required for manual versus automated segmentation of cervical spinous processes. For manual annotation, three annotators—two trained annotators and one expert spine surgeon—outlined the spinous processes on a predefined set of radiographs using a tablet interface. The total manual annotation time was determined by averaging the duration recorded across all three annotators. This process was divided into two stages: (1) annotation, measuring the average time required to trace the spinous processes, and (2) mask generation, recording the time needed to transform these annotations into binary masks using the color-based segmentation algorithm described in Section 2.1.

**FIGURE 9:**
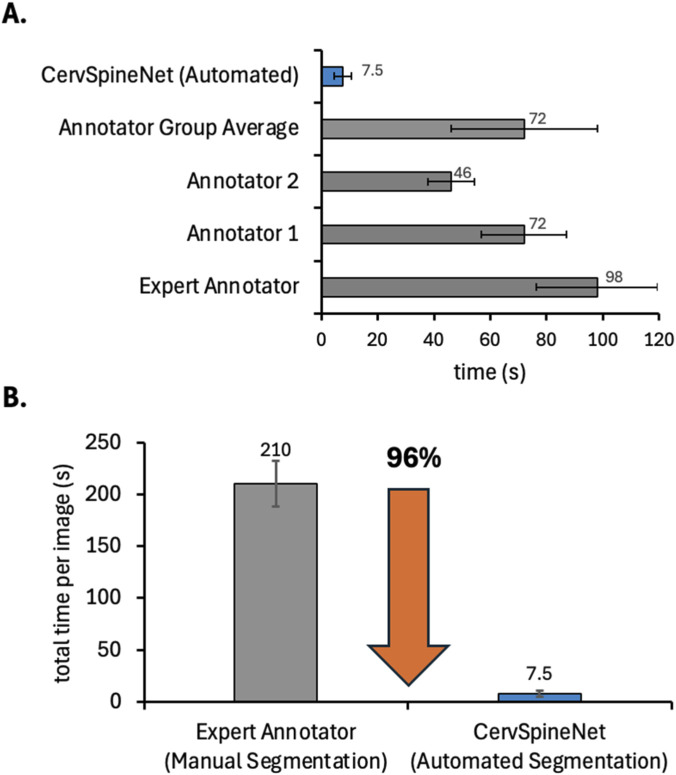
**(A)** Horizontal bar chart showing average annotation time per image for each manual annotator (Expert, Annotator 1, Annotator 2, and Annotator Group Average) compared with automated segmentation using CervSpineNet. Manual annotation required 46–98 s per image on average, whereas automated inference required only ∼7.5 s. **(B)** Vertical bar chart illustrating total segmentation time (annotation + mask generation) for manual versus automated approaches. Manual processing required approximately 3–4 min per image (mean = 210 s), while CervSpineNet produced binary masks in 5–10 s (mean = 7.5 s), representing a ∼96.43% reduction in total processing time. Error bars indicate standard deviation.

Figure 9 compares the time required for manual versus automated segmentation. Figure 9A summarizes the average annotation time per image for all manual annotators— two annotators and 1 expert—alongside the automated CervSpineNet model. Manual annotation times ranged from 46 to 98 s, with an overall average of 72 s, whereas CervSpineNet produced segmentations in only ∼7.5 s per image. Figure 9B shows total processing time, combining annotation and mask-generation stages. Manual processing required approximately 3–4 min per image (mean = 210 s). In contrast, automated segmentation using CervSpineNet took only 5–10 s (mean = 7.5 s), corresponding to an overall ∼96.43% reduction in total processing time.

Moreover, for inter-rater variability and to compare the model performance to human variability, we selected three of the test set images that the expert and trained annotators had already annotated and compared those segmentations to the masks predicted by the hybrid model using standard overlap and boundary metrics: Dice coefficient, Intersection-over-Union (IoU), 95th-percentile Hausdorff distance (HD95, in pixels), structural similarity index (SSIM), and volumetric similarity (VS).

Human–human agreement was high: expert vs. human 1 and expert vs. human two reached mean Dice scores of 0.91 (IoU ≈ 0.83–0.84, HD95 ≈ 6.3 pixels, SSIM ≈ 0.97, VS ≈ 0.96–0.98), while human 1 vs. human 2 yielded a similar Dice of 0.92 (IoU 0.85, HD95 6.24 pixels, SSIM 0.97, VS 0.98). On the same images, the proposed model reached a Dice of 0.92 vs. the expert (0.924), with higher IoU (0.86), lower HD95 (5.71 pixels), SSIM of 0.98, and VS of 0.99. Model vs. R1 and model vs. R2 comparisons were also in the same range: Dice ≈ 0.92, IoU ≈ 0.86, HD95 ≈ 6.8 and 5.5 pixels, SSIM ≈ 0.98. These results collectively indicate that model segmentations are as consistent with the expert as those of the additional human raters, and considering boundary accuracy, HD95 is slightly better than the average human–human variability on this sample, though we acknowledge this was based on three test cases.

## 4 Discussion

This section interprets the results of the study in the context of existing literature, emphasizing the methodological advances, clinical relevance, and potential impact of the developed CervSpineNet model. The discussion also outlines the limitations of the current work and identifies future directions for improving performance and generalizability.

### 4.1 Overview and model interpretation

This study introduces an automated segmentation framework for delineating cervical spinous processes from lateral spine radiographs. Because no public dataset existed for this task and manual annotation is labor-intensive, both a curated dataset and a novel hybrid model, CervSpineNet, were developed. Early zero-shot testing with standard segmentation models produced inconsistent and inaccurate delineations, confirming the need for a dedicated dataset capable of capturing the subtle morphology of spinous processes.

The CervSpineNet combines a Vision Transformer (ViT-B) encoder for global contextual modeling with a U-Net–style convolutional decoder for fine-grained boundary reconstruction (Chen J. et al., 2024; Al-Antari et al., 2025). This hybrid design maintains full spatial resolution by remaining fully convolutional, ensuring that predicted masks match input dimensions—a practical advantage for radiological workflows. Integrated residual and squeeze-and-excitation blocks selectively enhance task-relevant features and stabilize training. Collectively, these design elements allow the model to generate binary masks that closely align with ground truth and exhibit lower structural error, as confirmed across all dataset variants.

Although CervSpineNet follows the overall concept of a transformer–CNN hybrid, its architecture is specifically tailored to the demands of cervical spine X-ray segmentation. We decouple the SAM ViT-B image encoder and fine-tune it on 1,024 × 1,024 radiographs, leveraging its large-scale pretraining to capture long-range anatomical context along the full C1–C7 column. On top of these rich features, we employ a compact U-Net style decoder with residual and squeeze-and-excitation blocks, sharpening local boundaries and preserving thin, curved spinous process shapes. Training is driven by a compound loss that combines Dice overlap, Focal Tversky, Hausdorff distance–transform and SSIM terms to jointly encourage correct vertebral coverage, suppression of false positives and accurate contour geometry. In our experiments (Section 3.1, Tables 1–3), this SAM-based hybrid consistently outperforms standard U-Net, DeepLabV3+, a fine-tuned full SAM model and a text-guided SegFormer, indicating that the proposed combination of encoder, decoder and loss function confers a tangible advantage for this specific small-structure segmentation task.

Overall, the architectural ablations demonstrate that both encoder choice and decoder design substantially influence segmentation performance, albeit in complementary ways. Enhancing the decoder with residual and squeeze-and-excitation blocks consistently improves overlap, volumetric, and boundary metrics, indicating that richer decoding capacity is critical for refining fine spinal structures and suppressing noise. In contrast, replacing a purely convolutional encoder with a ViT-B transformer encoder yields the largest performance gains—particularly for boundary accuracy—highlighting the importance of long-range contextual reasoning and global shape modeling in cervical spine anatomy. Together, these results validate the design of CervSpineNet: the combination of a transformer-based encoder with an enriched decoder provides measurable advantages, while simpler components remain competitive options for lower-complexity or resource-constrained settings.

Loss ablations similarly show that region-based and structure-aware supervision outperforms Dice alone. Adding either Focal Tversky or SSIM to Dice produces consistent improvements in Dice, IoU, and SSIM, underscoring the value of class-imbalance handling and structural consistency for stable mask learning. The Dice + SSIM configuration is particularly effective for overlap and volumetric similarity, reflecting the benefit of enforcing local textural coherence in postoperative spine X-rays. These trends support our use of a compound loss: Dice and FT promote reliable region overlap and recall, SSIM preserves fine anatomical detail, and the HD95 surrogate sharpens boundaries by explicitly penalizing outlier deviations.

### 4.2 Performance evaluation and clinical relevance

Across the original, CLAHE-enhanced, and augmented datasets, CervSpineNet consistently outperformed CNN-based and transformer-based baselines on Dice, IoU, SSIM, HD95, and volumetric similarity metrics. Statistical testing confirmed that these improvements were significant, highlighting the robustness and reproducibility of the approach. Notably, the model accurately segmented difficult lower cervical levels (C6–C7), which are often challenging even for experts, underscoring its ability to generalize to low-contrast or ambiguous regions.

To the best of our knowledge, this work provides the first public dataset and segmentation framework dedicated to 2D X-ray–based cervical spinous process delineation. Accurate and efficient segmentation of these structures has broad clinical value. In Anterior Cervical Discectomy and Fusion (ACDF), precise localization of spinous processes supports surgical planning and fusion evaluation (Neifert et al., 2020; Martin et al., 2025). Beyond ACDF, automatic spinous process segmentation could aid in the assessment of vertebral alignment after trauma, the monitoring of spinal deformities such as scoliosis or kyphosis, and image-guided navigation during minimally invasive procedures.

From a workflow standpoint, CervSpineNet offers substantial efficiency gains. It reduces average manual segmentation time from approximately 3–4 min per image to 5–10 s, representing a ∼96% time reduction. The lightweight architecture (∼345 MB) and CPU-compatible inference make the model suitable for clinical integration without specialized hardware. Collectively, these features position CervSpineNet as a practical, reproducible tool for advancing AI-assisted cervical spine imaging and analysis.

### 4.3 Limitations and future work

Despite strong performance, several limitations warrant consideration. First, training the hybrid architecture is more computationally demanding than lighter CNN baselines due to the transformer encoder and multi-stage decoding. Model performance may also be sensitive to threshold selection, class imbalance, and annotation noise, and the fixed-size image resizing may introduce subtle aspect-ratio bias. Although CervSpineNet performs well on internal data, external validation across different scanners, institutions, and acquisition protocols remains necessary to assess generalization and potential domain shift. Additionally, while we report strong segmentation metrics (Dice, IoU, HD95, etc.), we did not directly evaluate downstream clinical indices—such as spinous-process–derived angular measures or deformity parameters. Establishing links between CervSpineNet’s segmentations and clinically meaningful quantitative metrics will be essential for demonstrating translational impact.

Future optimization efforts should investigate encoder freezing, mixed-precision training, gradient checkpointing, or knowledge distillation to reduce computational burden. Boundary accuracy may be further improved through boundary-aware decoders, attention-gated skip connections, or uncertainty-based quality control mechanisms. Expanding the dataset to incorporate diverse imaging protocols, patient populations, and post-operative presentations will enhance robustness. Extending the framework to multi-view radiographs or 3D modalities (CT, MRI) may also enrich anatomical representation and support more complex clinical tasks.

Prospective clinical validation and workflow-integrated deployment studies will be critical for determining real-world utility in surgical planning, postoperative monitoring, and routine spine care. Beyond surgical settings, automated spinous-process segmentation may facilitate longitudinal deformity surveillance, trauma assessment, and AI-assisted spine navigation. These directions position CervSpineNet as a promising foundation for scalable, clinically relevant spine-imaging solutions.

## 5 Conclusion

This study presents CervSpineNet, a hybrid transformer–CNN framework for automated segmentation of cervical spinous processes on lateral X-ray images, along with the first curated dataset developed specifically for this task. By coupling a ViT-B encoder that captures global anatomical context with a lightweight convolutional decoder optimized for fine structural reconstruction, CervSpineNet achieves strong performance, with mean Dice scores exceeding 0.93 and SSIM values above 0.98 across multiple dataset variants. Statistical comparisons demonstrate significant improvements over established baselines, and the model reduces manual annotation time by approximately 96%, producing accurate binary masks within 5–10 s on standard clinical hardware.

With its high accuracy, compact computational footprint, and openly accessible dataset, CervSpineNet offers a practical and reproducible foundation for future clinical integration and methodological research. The framework has potential applications in spine imaging, surgical planning, postoperative monitoring, and quantitative radiology, and it provides a scalable platform for advancing automated analysis of cervical spine anatomy.

## Data Availability

The original contributions presented in the study are included in the article/supplementary material, further inquiries can be directed to the corresponding author.
